# Supplementary material for: Hierarchical TAF1-dependent co-translational assembly of the basal transcription factor TFIID
Source: Nat Struct Mol Biol. 2023 Jun 29;30(8):1141–52. doi: 10.1038/s41594-023-01026-3 (PMC10442232; doi:10.1038/s41594-023-01026-3)
Supplement: Supplementary file 1 — Supplementary Tables 1–3 [file 41594_2023_1026_MOESM1_ESM.pdf]

nature structural & molecular biology

Article

https://doi.org/10.1038/s41594-023-01026-3

Hierarchical TAF1-dependent co-translational assembly of the basal transcription factor TFIID

In the format provided by the authors and unedited

Supplementary Table 1: summary of TFIID co-translational assembly events. Colors correspond to subunits color-code used in this work (related to Figure 1).

nascent protein	co-translational interaction partner
TAF1 ♦	TAF2 ♦
TAF1 ♦	TAF4 ♦
TAF1 ♦	TAF5 ♦
TAF1 ♦	TAF6 ♦
TAF1 ♦	TAF7 ♦
TAF1 ♦	TAF8 ♦
TAF1 ♦	TAF10 ♦
TAF1 ♦	TAF12 ♦
TAF1 ♦	TBP ♦
TAF2 ♦	TAF8 ♦
TAF3 ♦	TAF10 ♦
TAF4 ♦	TAF12 ♦
TAF6 ♦	TAF9 ♦
TAF6 ♦	TAF5 ♦
TAF7 ♦	TAF1 ♦
TAF8 ♦	TAF2 ♦
TAF8 ♦	TAF10 ♦
TAF9 ♦	TAF6 ♦
TAF11 ♦	TAF13 ♦
TAF13 ♦	TAF11 ♦

Supplementary Table 2: X-linking MS combined table (related to Figure 5)

Summary of TAF1-centered crosslinking-MS metanalysis performed on seven distinct datasets from three different groups: Patel et al., 2018 (TFIID), Scheer et al., 2021 (TFIID), Chen et al., 2021 (TFIID incorporated in different preinitiation complexes: cPICscp, cPICpuma, mPICscp, hPICscp, p53hPIChdm2). Only crosslinks found in more than one dataset were considered.

TAF1 interprotein crosslinks

Protein 1	Protein 2	Position 1	Position 2	Protein 1	Protein 2	Position 1	Position 2
TAF1	TAF11	249	95	TAF1	TAF6	378	357
TAF1	TAF11	249	97	TAF1	TAF6	424	342
TAF1	TAF11	249	105	TAF1	TAF6	427	342
TAF1	TAF11	249	197	TAF1	TAF6	427	287
TAF1	TAF11	249	197	TAF1	TAF7	622	5
TAF1	TAF13	249	101	TAF1	TAF7	622	291
TAF1	TAF13	249	101	TAF1	TAF7	674	40
TAF1	TAF2	544	383	TAF1	TAF7	674	40
TAF1	TAF2	544	788	TAF1	TAF7	817	5
TAF1	TAF2	562	788	TAF1	TAF7	819	5
TAF1	TAF2	576	384	TAF1	TAF7	832	5
TAF1	TAF2	576	565	TAF1	TAF7	889	164
TAF1	TAF2	701	513	TAF1	TAF7	906	164
TAF1	TAF2	710	595	TAF1	TAF7	906	167
TAF1	TAF2	945	513	TAF1	TAF7	1127	164
TAF1	TAF5	370	318	TAF1	TAF7	1187	164
TAF1	TAF6	330	65	TAF1	TAF7	1201	155
TAF1	TAF6	330	196	TAF1	TAF7	1201	153
TAF1	TAF6	330	158	TAF1	TAF7	1208	167
TAF1	TAF6	335	196	TAF1	TAF8	427	178
TAF1	TAF6	370	196	TAF1	TAF9	330	24
TAF1	TAF6	370	367	TAF1	TAF9	330	10
TAF1	TAF6	370	361	TAF1	TBP	168	243
TAF1	TAF6	378	361	TAF1	TBP	170	333
TAF1	TAF6	378	361	TAF1	TBP	1009	181
TAF1	TAF6	378	367				

TAF1 intraprotein crosslinks

Protein 1	Protein 2	Position 1	Position 2	Protein 1	Protein 2	Position 1	Position 2
TAF1	TAF1	330	370	TAF1	TAF1	1009	1046
TAF1	TAF1	335	370	TAF1	TAF1	1009	1127
TAF1	TAF1	367	370	TAF1	TAF1	1009	1111
TAF1	TAF1	527	531	TAF1	TAF1	1046	1063
TAF1	TAF1	531	536	TAF1	TAF1	1046	1201
TAF1	TAF1	531	1436	TAF1	TAF1	1111	1127
TAF1	TAF1	531	976	TAF1	TAF1	1111	1201
TAF1	TAF1	531	544	TAF1	TAF1	1111	1187
TAF1	TAF1	536	544	TAF1	TAF1	1112	1187
TAF1	TAF1	536	549	TAF1	TAF1	1112	1201
TAF1	TAF1	536	576	TAF1	TAF1	1112	1127
TAF1	TAF1	544	943	TAF1	TAF1	1112	1177

TAF1	TAF1	544	1063	TAF1	TAF1	1112	1208
TAF1	TAF1	544	1009	TAF1	TAF1	1117	1127
TAF1	TAF1	544	576	TAF1	TAF1	1127	1208
TAF1	TAF1	544	549	TAF1	TAF1	1127	1187
TAF1	TAF1	544	710	TAF1	TAF1	1127	1166
TAF1	TAF1	549	706	TAF1	TAF1	1127	1201
TAF1	TAF1	549	707	TAF1	TAF1	1166	1187
TAF1	TAF1	549	1063	TAF1	TAF1	1187	1208
TAF1	TAF1	549	705	TAF1	TAF1	1201	1208
TAF1	TAF1	576	701	TAF1	TAF1	1201	1222
TAF1	TAF1	576	1063	TAF1	TAF1	1240	1255
TAF1	TAF1	576	943	TAF1	TAF1	1240	1244
TAF1	TAF1	576	945	TAF1	TAF1	1249	1255
TAF1	TAF1	611	832	TAF1	TAF1	1249	1261
TAF1	TAF1	611	621	TAF1	TAF1	1261	1581
TAF1	TAF1	611	622	TAF1	TAF1	1305	1322
TAF1	TAF1	611	976	TAF1	TAF1	1305	1327
TAF1	TAF1	621	832	TAF1	TAF1	1317	1339
TAF1	TAF1	622	1111	TAF1	TAF1	1317	1327
TAF1	TAF1	641	674	TAF1	TAF1	1322	1327
TAF1	TAF1	701	710	TAF1	TAF1	1322	1344
TAF1	TAF1	701	707	TAF1	TAF1	1322	1339
TAF1	TAF1	705	1063	TAF1	TAF1	1327	1344
TAF1	TAF1	705	710	TAF1	TAF1	1327	1535
TAF1	TAF1	705	707	TAF1	TAF1	1327	1487
TAF1	TAF1	817	832	TAF1	TAF1	1327	1329
TAF1	TAF1	817	1004	TAF1	TAF1	1339	1581
TAF1	TAF1	819	832	TAF1	TAF1	1339	1347
TAF1	TAF1	819	1004	TAF1	TAF1	1344	1487
TAF1	TAF1	831	1205	TAF1	TAF1	1344	1354
TAF1	TAF1	899	1208	TAF1	TAF1	1344	1399
TAF1	TAF1	899	1187	TAF1	TAF1	1353	1399
TAF1	TAF1	899	1201	TAF1	TAF1	1372	1487
TAF1	TAF1	906	1208	TAF1	TAF1	1372	1555
TAF1	TAF1	906	1201	TAF1	TAF1	1412	1419
TAF1	TAF1	906	1187	TAF1	TAF1	1412	1535
TAF1	TAF1	922	1009	TAF1	TAF1	1412	1542
TAF1	TAF1	922	1201	TAF1	TAF1	1414	1419
TAF1	TAF1	943	971	TAF1	TAF1	1415	1535
TAF1	TAF1	943	967	TAF1	TAF1	1415	1542
TAF1	TAF1	945	976	TAF1	TAF1	1419	1534
TAF1	TAF1	967	976	TAF1	TAF1	1419	1542
TAF1	TAF1	971	979	TAF1	TAF1	1419	1535
TAF1	TAF1	971	986	TAF1	TAF1	1454	1534
TAF1	TAF1	976	979	TAF1	TAF1	1454	1535
TAF1	TAF1	976	986	TAF1	TAF1	1463	1535
TAF1	TAF1	979	987	TAF1	TAF1	1463	1534

TAF1	TAF1	979	986	TAF1	TAF1	1480	1493
TAF1	TAF1	986	1009	TAF1	TAF1	1480	1487
TAF1	TAF1	986	1046	TAF1	TAF1	1482	1487
TAF1	TAF1	986	1127	TAF1	TAF1	1482	1493
TAF1	TAF1	986	1001	TAF1	TAF1	1487	1493
TAF1	TAF1	1001	1009	TAF1	TAF1	1493	1559
TAF1	TAF1	1001	1018	TAF1	TAF1	1534	1542
TAF1	TAF1	1004	1018	TAF1	TAF1	1542	1581
TAF1	TAF1	1009	1063	TAF1	TAF1	1561	1622
TAF1	TAF1	1009	1006				

Supplementary Table 3: Antibodies used in the paper and references

Target/Name	Clonality	ID code/Clone (Supplier/Lot#/Ref.)	Application	Dilution
TAF1	rabbit pAb	ab188427 (Abcam, lot GR178560-25)	IF	1:1000
TAF1	rabbit pAb	ab264327 (Abcam, lot 1017147-1)	IP, WB	1:2000 (WB)
TAF2	rabbit pAb	#3038 (IGBMC, Trowitzsch et al., 2015)	IP	NA
TAF4	mouse mAb	32TA 2B9 (IGBMC, Mohan et al., 2003)	IP, IF, WB	2 ug/mL (purified Ab for IF), 1:500 (WB)
TAF5	mouse mAb	1TA 1C2 (IGBMC, Dantonel et al., 1997)	WB	1:500
TAF6	mouse mAb	25TA 2G7 (IGBMC, Dantonel et al., 1997)	WB	1:500
TAF6	rabbit pAb	A301-275A (Bethyl, lot A301-275A-1)	RIP	NA
TAF7	rabbit pAb	#3475 (IGBMC, Bardot et al., 2017)	IP, IF	1:250 (IF)
TAF7	mouse mAb	31TA 2C12 (IGBMC, present work)	RIP	NA
TAF7	mouse mAb	19TA 2C7 (IGBMC, Lavigne et al., 1996)	WB	1:500
TAF8	rabbit pAb	#3478 (IGBMC, Bardot et al., 2017)	WB	1:1000
TAF9	goat pAb	sc-1248 (Santa Cruz Biotechnology, lot F2806)	WB	1:400
TAF10	mouse mAb	23TA 1H8 (IGBMC, Soutoglou et al., 2005)	IP, RIP	NA
TAF10	mouse mAb	6TA 2B11 (IGBMC, Wieczorek et al., 1998)	RIP, IF, WB	3 ug/mL (purified Ab for IF), 1:1000 (WB)
TAF11	mouse mAb	15TA 2B4 (IGBMC, Gupta et al., 2017)	IP	NA
TAF12	mouse mAb	22TA 2A1 (IGBMC, Malecova et al., 2016)	WB	1:500
TAF13	mouse mAb	16TA 3C12 (IGBMC, Mengus et al., 1995)	WB	1:500
TBP	mouse mAb	3TF1 3G3 (IGBMC, Brou et al., 1993)	WB, IF	2 ug/mL (purified Ab for IF), 1:1000 (WB)
SUPT7L	rabbit pAb	A302-803A (Bethyl, lot A302-803A-1)	IF	1:500
lamin A/C	mouse mAb	sc-7292 (Santa Cruz Biotechnology, lot L2314)	WB	1:250
GST	mouse mAb	15TF2 1D10 (Creative Biolabs)	IP	NA
GAPDH	rabbit mAb	14C10 (Cell Signaling Technology, lot 14)	WB	1:1000
histone H3	rabbit pAb	ab1791 (Abcam, lot GR3198176-1)	WB	1:10000
AF488 goat anti-mouse IgG	goat pAb	A11001 (Life Technologies, lot 1259373)	IF	1:3000
AF488 goat anti-rabbit IgG	goat pAb	A11008 (Life Technologies, lot 1829924)	IF	1:3000
AF(Plus)647 goat anti-mouse IgG	goat pAb	A32728 (Life Technologies, lot WK331591)	IF	1:3000
HRP goat anti-mouse IgG	goat pAb	115-036-071 (Jackson ImmunoResearch, lot 160707)	WB	1:10000
HRP goat anti-rabbit IgG	goat pAb	111-035-144 (Jackson ImmunoResearch, lot 161546)	WB	1:10000

Antibody supplementary references

Bardot, P., Vincent, S.D., Fournier, M., Hubaud, A., Joint, M., Tora, L., Pourquié, O., 2017. The TAF10-containing TFIID and SAGA transcriptional complexes are dispensable for early somitogenesis in the mouse embryo. Development 144, 3808–3818. https://doi.org/10.1242/dev.146902

Brou, C., Chaudhary, S., Davidson, I., Lutz, Y., Wu, J., Egly, J.M., Tora, L., Chambon, P., 1993. Distinct TFIID complexes mediate the effect of different transcriptional activators. EMBO J 12, 489–499. https://doi.org/10.1002/j.1460-2075.1993.tb05681.x

Dantonel, J.C., Murthy, K.G., Manley, J.L., Tora, L., 1997. Transcription factor TFIID recruits factor CPSF for formation of 3' end of mRNA. Nature 389, 399–402. https://doi.org/10.1038/38763

Gupta, K., Watson, A.A., Baptista, T., Scheer, E., Chambers, A.L., Koehler, C., Zou, J., Obong-Ebong, I., Kandiah, E., Temblador, A., Round, A., Forest, E., Man, P., Bieniossek, C., Laue, E.D., Lemke, E.A., Rappsilber, J., Robinson, C.V., Devys, D., Tora, L., Berger, I., 2017. Architecture of TAF11/TAF13/TBP complex suggests novel regulation properties of general transcription factor TFIID. Elife 6, e30395. https://doi.org/10.7554/eLife.30395

Lavigne, A.C., Mengus, G., May, M., Dubrovskaya, V., Tora, L., Chambon, P., Davidson, I., 1996. Multiple interactions between hTAFII55 and other TFIID subunits. Requirements for the formation of stable ternary complexes between hTAFII55 and the TATA-binding protein. J Biol Chem 271, 19774–19780. https://doi.org/10.1074/jbc.271.33.19774

Malecova, B., Dall'Agnese, A., Madaro, L., Gatto, S., Coutinho Toto, P., Albini, S., Ryan, T., Tora, L., Puri, P.L., 2016. TBP/TFIID-dependent activation of MyoD target genes in skeletal muscle cells. eLife 5, e12534. https://doi.org/10.7554/eLife.12534

Mengus, G., May, M., Jacq, X., Staub, A., Tora, L., Chambon, P., Davidson, I., 1995. Cloning and characterization of hTAFII18, hTAFII20 and hTAFII28: three subunits of the human transcription factor TFIID. EMBO J 14, 1520–1531.

Mohan, W.S., Scheer, E., Wendling, O., Metzger, D., Tora, L., 2003. TAF10 (TAFII30) Is Necessary for TFIID Stability and Early Embryogenesis in Mice. Molecular and Cellular Biology 23, 4307–4318. https://doi.org/10.1128/MCB.23.12.4307-4318.2003

Soutoglou, E., Demény, M.A., Scheer, E., Fienga, G., Sassone-Corsi, P., Tora, L., 2005. The Nuclear Import of TAF10 Is Regulated by One of Its Three Histone Fold Domain-Containing Interaction Partners. Molecular and Cellular Biology 25, 4092–4104. https://doi.org/10.1128/MCB.25.10.4092-4104.2005

Trowitzsch, S., Viola, C., Scheer, E., Conic, S., Chavant, V., Fournier, M., Papai, G., Ebong, I.-O., Schaffitzel, C., Zou, J., Haffke, M., Rappsilber, J., Robinson, C.V., Schultz, P., Tora, L., Berger, I., 2015. Cytoplasmic TAF2–TAF8–TAF10 complex provides evidence for nuclear holo–TFIID assembly from preformed submodules. Nat Commun 6, 6011. https://doi.org/10.1038/ncomms7011

Wieczorek, E., Brand, M., Jacq, X., Tora, L., 1998. Function of TAF(II)-containing complex without TBP in transcription by RNA polymerase II. Nature 393, 187–191. https://doi.org/10.1038/30283
