# Supplementary material for: Hierarchical TAF1-dependent co-translational assembly of the basal transcription factor TFIID
Source: Nat Struct Mol Biol. 2023 Jun 29;30(8):1141–52. doi: 10.1038/s41594-023-01026-3 (PMC10442232; doi:10.1038/s41594-023-01026-3)
Supplement: Supplementary file 2 — Reporting Summary [file 41594_2023_1026_MOESM2_ESM.pdf]

nature portfolio

Corresponding author(s): Laszlo TORA

Last updated by author(s): Apr 20, 2023

Reporting Summary

Nature Portfolio wishes to improve the reproducibility of the work that we publish. This form provides structure for consistency and transparency in reporting. For further information on Nature Portfolio policies, see our Editorial Policies and the Editorial Policy Checklist.

Statistics

For all statistical analyses, confirm that the following items are present in the figure legend, table legend, main text, or Methods section.

n/a	Confirmed	
☐	☒	The exact sample size (*n*) for each experimental group/condition, given as a discrete number and unit of measurement
☐	☒	A statement on whether measurements were taken from distinct samples or whether the same sample was measured repeatedly
☐	☒	The statistical test(s) used AND whether they are one- or two-sided<br>*Only common tests should be described solely by name; describe more complex techniques in the Methods section.*
☒	☐	A description of all covariates tested
☒	☐	A description of any assumptions or corrections, such as tests of normality and adjustment for multiple comparisons
☐	☒	A full description of the statistical parameters including central tendency (e.g. means) or other basic estimates (e.g. regression coefficient) AND variation (e.g. standard deviation) or associated estimates of uncertainty (e.g. confidence intervals)
☐	☒	For null hypothesis testing, the test statistic (e.g. *F*, *t*, *r*) with confidence intervals, effect sizes, degrees of freedom and *P* value noted<br>*Give P values as exact values whenever suitable.*
☒	☐	For Bayesian analysis, information on the choice of priors and Markov chain Monte Carlo settings
☒	☐	For hierarchical and complex designs, identification of the appropriate level for tests and full reporting of outcomes
☒	☐	Estimates of effect sizes (e.g. Cohen's *d*, Pearson's *r*), indicating how they were calculated

Our web collection on statistics for biologists contains articles on many of the points above.

Software and code

Policy information about availability of computer code

Data collection	RT-qPCR data acquisition was performed using LightCycler® 480 Software (Roche, version 1.5.0). Microscopy data collection was performed using MetaMorph® software (Molecular Devices). Western blot images were acquired using a ChemiDoc Touch system (BioRad).
Data analysis	Microscopy data processing and analysis was performed in Fiji (ImageJ version 2.9.0) and CellProfiler (version 4.2.1). Chromatic shift correction was performed using Chromagnon (version v0.90). Western blot images were processed in ImageLab software (BioRad, version 6.0). Mass spectrometry protein identification and analysis was performed using SequestHT in the Proteome Discoverer software (Thermo Fischer Scientific, versions 2.4-2.5). Structural analysis and visualisation was performed in UCSF ChimeraX software (version 1.4). Data rearrangement and plotting was performed in RStudio (R version 4.2.1) with tidyverse package (version 1.3.2). Figures were prepared using Inkscape (version 1.2). This work did not generate any original code relevant for data analysis.

For manuscripts utilizing custom algorithms or software that are central to the research but not yet described in published literature, software must be made available to editors and reviewers. We strongly encourage code deposition in a community repository (e.g. GitHub). See the Nature Portfolio guidelines for submitting code & software for further information.

Data

Policy information about availability of data

All manuscripts must include a data availability statement. This statement should provide the following information, where applicable:

- Accession codes, unique identifiers, or web links for publicly available datasets
- A description of any restrictions on data availability
- For clinical datasets or third party data, please ensure that the statement adheres to our policy

AlphaFold protein structure database (https://alphafold.ebi.ac.uk/) was used to download human TAF1 structural prediction file (accession P21675). Human FASTA database downloaded from UniProt (reviewed, releases 2021_06_03 and 2022_02_21, https://www.uniprot.org/) was used as reference database for mass-spectrometry protein identification. LC-MS/MS data have been deposited at PRIDE repository with the identifier PXD036358 and are publicly available as of the date of publication. Ribosome footprinting data plot was obtained from RiboCrypt browser (https://ribocrypt.org/) using human TAF1 transcript accession ENST00000373790 selecting 'all_merged-Homo_sapiens' as experiment. Any additional information required to reanalyze the data reported in this paper is available from the lead contact (László Tora, laszlo@igbmc.fr) upon request. This paper contains Source Data files.

Human research participants

Policy information about studies involving human research participants and Sex and Gender in Research.

Reporting on sex and gender	Not relevant to the study.
Population characteristics	Not relevant to the study.
Recruitment	Not relevant to the study.
Ethics oversight	Not relevant to the study.

Note that full information on the approval of the study protocol must also be provided in the manuscript.

Field-specific reporting

Please select the one below that is the best fit for your research. If you are not sure, read the appropriate sections before making your selection.

☒ Life sciences ☐ Behavioural & social sciences ☐ Ecological, evolutionary & environmental sciences

For a reference copy of the document with all sections, see nature.com/documents/nr-reporting-summary-flat.pdf

Life sciences study design

All studies must disclose on these points even when the disclosure is negative.

Sample size	We planned our experiments in analogy to previously published articles (such as Kamenova et al. 2018). Conclusions drawn in the study were derived by several alternative experimental approaches. No sample-size calculation was performed.
Data exclusions	No data were excluded from the analyses.
Replication	RIP-qPCR data reported in all the figures were calculated from 2 or 3 biological replicates (specified in figure legends). Western blot of TAF6 and TAF7 antibodies validation for RIP assays shown in Extended Data Figure 1e was performed once. Cell-fractionation upon TAF1 knockdown followed by protein repartition analysis by western blot shown in Figure 6a was performed two times and the results confirmed by mass spectrometry on immunopurified endogenous TFIID subunits shown in Figure 6b. The same experiment upon TAF4 and TAF7 knockdown in Extended Data Figure 6a was performed once. The protocols/experiments provided in the manuscript were reproducible.
Randomization	Randomization was not required for the biochemical/molecular experiments. Images for microscopy analyses (immunofluorescence + smFISH) were randomized before manual investigation (blinding). Other analyses were less prone to direct human-based biases thanks to instrument readouts, therefore randomization was not performed.
Blinding	The investigator was blinded to samples identity during microscopy data analysis concerning the manual detection of co-localized signals to limit human biases. Other experiments were less prone to direct human-based biases thanks to instrument readouts, therefore blinding was not practiced.

Reporting for specific materials, systems and methods

We require information from authors about some types of materials, experimental systems and methods used in many studies. Here, indicate whether each material, system or method listed is relevant to your study. If you are not sure if a list item applies to your research, read the appropriate section before selecting a response.

Materials & experimental systems

n/a	Involved in the study
☐	☒ Antibodies
☐	☒ Eukaryotic cell lines
☒	☐ Palaeontology and archaeology
☒	☐ Animals and other organisms
☒	☐ Clinical data
☒	☐ Dual use research of concern

Methods

n/a	Involved in the study
☒	☐ ChIP-seq
☒	☐ Flow cytometry
☒	☐ MRI-based neuroimaging

Antibodies

Antibodies used

See also Supplementary File 1 for a full antibodies table information (with dilution).
TAF1 (Abcam, rabbit pAb, ab188427); TAF1 (Abcam, rabbit pAb, ab264327); TAF2 (IGBMC, rabbit pAb, #3038); TAF4 (IGBMC, mouse mAb, 32TA 2B9); TAF5 (IGBMC, mouse mAb, 1TA 1C2); TAF6 (IGBMC, mouse mAb, 25TA 2G7); TAF6 (Bethyl, rabbit pAb, A301-275A); TAF7 (IGBMC, rabbit pAb, #3475); TAF7 (IGBMC, mouse mAb); TAF7 (IGBMC, mouse mAb, 19TA 2C7); TAF8 (IGBMC, rabbit pAb, #3478); TAF9 (Santa Cruz Biotechnology, goat pAb, sc-1248); TAF10 (IGBMC, mouse mAb, 23TA 1H8); TAF10 (IGBMC, mouse mAb, 6TA 2B11); TAF11 (IGBMC, mouse mAb, 15TA 2B4); TAF12 (IGBMC, mouse mAb, 22TA 2A1); TAF13 (IGBMC, mouse mAb, 16TA 3C12); TBP (IGBMC, mouse mAb, 3TF1 3G3); SUPT7L (Bethyl, rabbit pAb, A302-803A); GST (Creative Biolabs, mouse mAb, 15TF2 1D10); lamin A/C (Santa Cruz Biotechnology, mouse mAb, sc-7292); GAPDH (Cell Signaling Technology, rabbit mAb, 14C10); histone H3 (Abcam, rabbit pAb, ab1791); AF488 goat anti-mouse IgG (Life Technologies, goat pAb, A11001); AF488 goat anti-rabbit IgG (Life Technologies, goat pAb, A11008); AF(Plus)647 goat anti-mouse IgG (Life Technologies, goat pAb, A32728); HRP goat anti-mouse IgG (Jackson ImmunoResearch, goat pAb, 115-036-071); HRP goat anti-rabbit IgG (Jackson ImmunoResearch, goat pAb, 111-035-144).

Validation

See also Supplementary File 1 for a full antibodies table information (with dilution).
Antibody validation references are provided along each antibody used in this study in Supplementary File 1. Also note that antibodies used in immunoprecipitation coupled to mass-spectrometry successfully enriched the target protein.
TAF1 (Abcam, ab188427): IF (https://www.abcam.com/products/primary-antibodies/taf1-antibody-ab188427.html)
TAF1 (Abcam, ab264327): IP, WB (https://www.abcam.com/products/primary-antibodies/taf1-antibody-ab264327.html)
TAF2 (IGBMC, #3038): IP (Trowitzsch et al., 2015)
TAF4 (IGBMC, 32TA 2B9): IP, WB (Mohan et al., 2003)
TAF5 (IGBMC, 1TA 1C2): WB (Dantonel et al., 1997)
TAF6 (IGBMC, 25TA 2G7): WB (Dantonel et al., 1997)
TAF6 (Bethyl, A301-275A): IP (https://www.thermofisher.com/antibody/product/TAF6-Antibody-Polyclonal/A301-275A)
TAF7 (IGBMC, #3475): IP (Bardot et al., 2017)
TAF7 (IGBMC, 31TA 2C12): IP (present work)
TAF7 (IGBMC, 19TA 2C7): WB, IP (Lavigne et al., 1996)
TAF8 (IGBMC, #3478): WB (Bardot et al., 2017)
TAF9 (Santa Cruz Biotechnology, sc-1248): WB (https://datasheets.scbt.com/sc-1248.pdf)
TAF10 (IGBMC, 23TA 1H8): IP (Soutoglou et al., 2005)
TAF10 (IGBMC, 6TA 2B11): WB (Wieczorek et al., 1998), IF (Soutoglou et al., 2005)
TAF11 (IGBMC, 15TA 2B4): IP (Gupta et al., 2017)
TAF12 (IGBMC, 22TA 2A1): WB (Malecova et al., 2016)
TAF13 (IGBMC, 16TA 3C12): WB (Mengus et al., 1995)
TBP (IGBMC, 3TF1 3G3): WB (Brou et al., 1993)
SUPT7L (Bethyl, A302-803A): WB, IHC (https://fortis-datasheets.s3.us-east-2.amazonaws.com/A302-803A-1.pdf)
GST (Creative Biolabs, 15TF2 1D10): IP (https://www.antibody-creativebiolabs.com/anti-hpgds-antibody-cblg1-2281-87009.htm)
lamin A/C (Santa Cruz Biotechnology, sc-7292): WB (https://www.scbt.com/p/lamin-a-c-antibody-636)
GAPDH (Cell Signaling Technology, 14C10): WB, IF, IHC (https://www.cellsignal.com/products/primary-antibodies/gapdh-14c10-rabbit-mab/2118)
histone H3 (Abcam, ab1791): WB, IP, ChIP, IF (https://www.abcam.com/products/primary-antibodies/histone-h3-antibody-nuclear-marker-and-chip-grade-ab1791.html)

Eukaryotic cell lines

Policy information about cell lines and Sex and Gender in Research

Cell line source(s)

HeLa cells were obtained from IGBMC cell culture facility. HeLa Flp-In/T-REx cells for the generation of GFP-TAF fusion expressing cell lines were not from a commercial source and they were described in van Nuland et al., 2013 (MCB, doi:10.1128/MCB.01742-12) and Antonova et al., 2018 (NSMB, doi:10.1038/s41594-018-0156-z). The resulting engineered GFP-fusion cell lines were described in Antonova, 2020 (doi:10.33540/207). The E14 mouse embryonic stem cells (ES Parental cell line E14Tg2a.4) were obtained from Mutant Mouse Resource and Research Center (MMRRC) (Citation ID:RRID:MMRRC_015890-UCD).

Authentication

HeLa cells were authenticated according to ATCC STR profiling. mESCs morphology was periodically controlled.

Mycoplasma contamination	All cell lines used in the study were tested for mycoplasma contamination by the IGBMC Cell Culture Facility and were negative.
Commonly misidentified lines (See ICLAC register)	No commonly misidentified cell lines were employed for the study.
