# Supplementary figures and images for: Hierarchical TAF1-dependent co-translational assembly of the basal transcription factor TFIID
Source: Nat Struct Mol Biol. 2023 Jun 29;30(8):1141–52. doi: 10.1038/s41594-023-01026-3 (PMC10442232; doi:10.1038/s41594-023-01026-3)

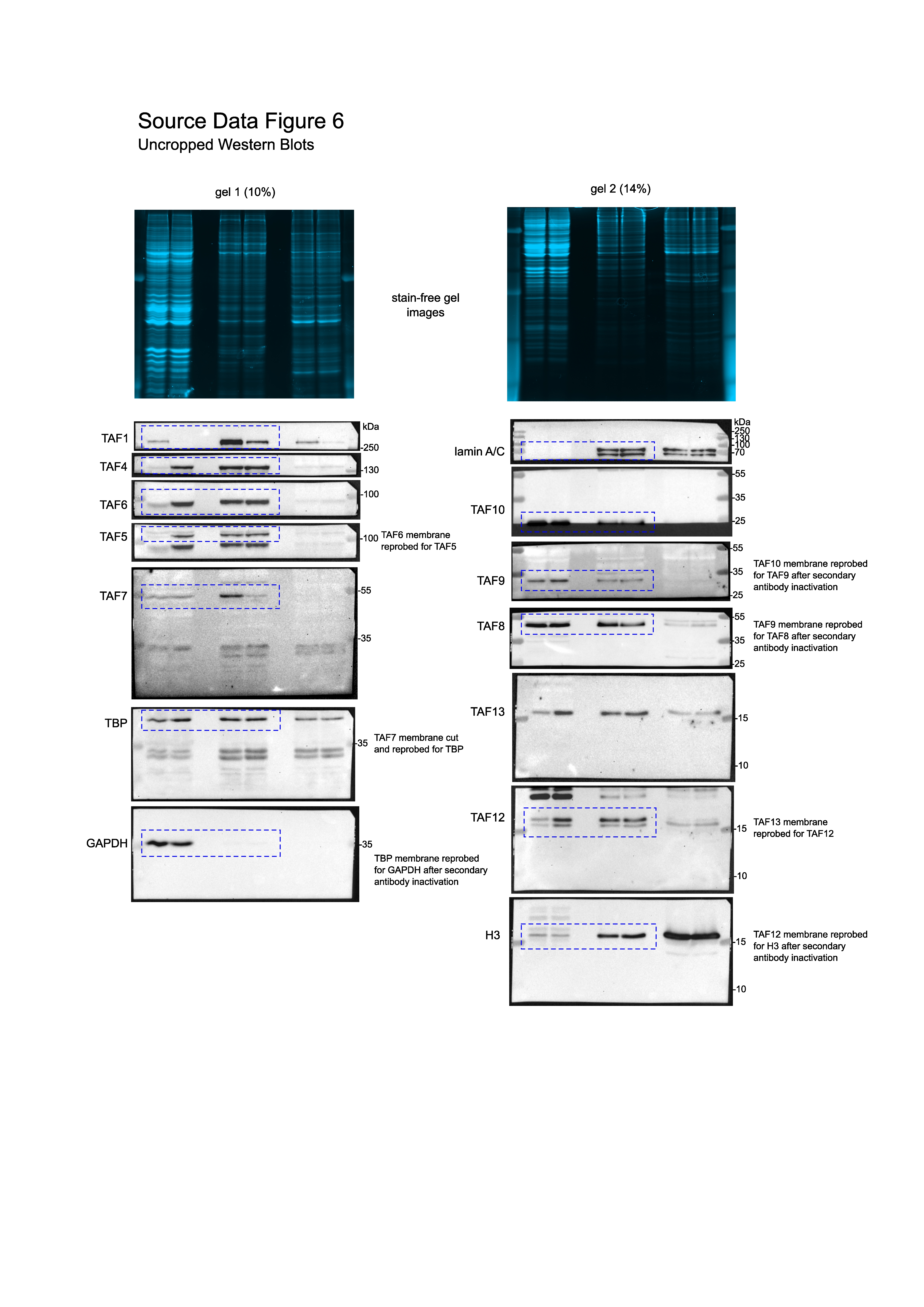

Supplement: Source Data Fig. 6 — Uncropped western blot images for Fig. 6 blots. [file 41594_2023_1026_MOESM10_ESM.jpg]

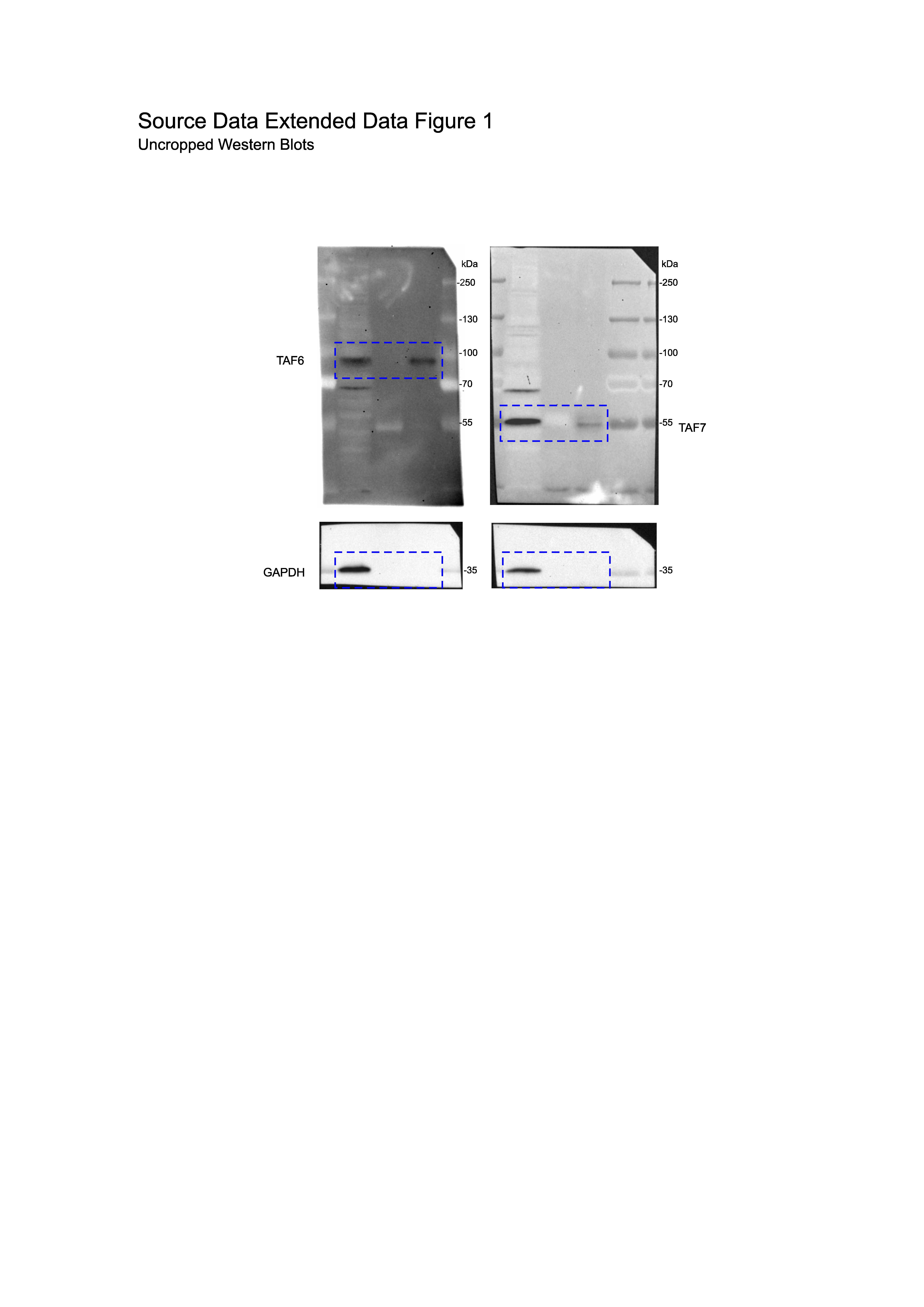

Supplement: Source Data Extended Data Fig. 1 — Uncropped western blot images for Extended Data Figure 1 blots. [file 41594_2023_1026_MOESM12_ESM.jpg]

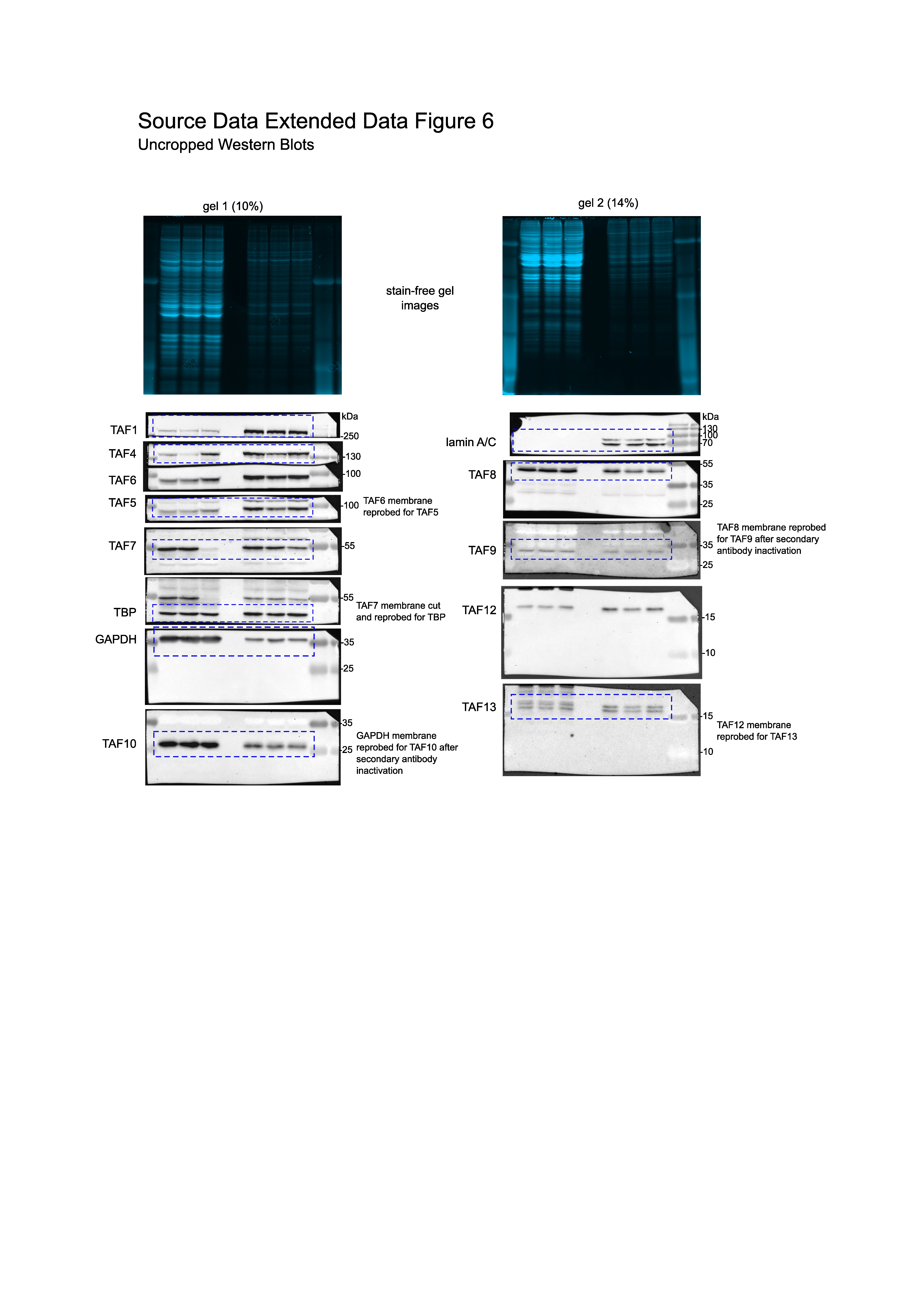

Supplement: Source Data Extended Data Fig. 6 — Uncropped western blot images for Extended Data Figure 6 blots. [file 41594_2023_1026_MOESM17_ESM.jpg]
